# The Week's Work

**Published:** 1910-09-17

**Authors:** 


### The Week's Work.

### THE HULL SANATORIUM.

Considerable agitation is going on at Hull in connection with the recent sanatorium scandal. It will be remembered by those who followed the case that the Corporation appointed a special investigation committee to inquire into the allegations. It is now stated that this committee has not gone fully into the question and that it was prejudiced. Seven of the members were also members of the hospital sub-committee, and this fact has given rise to a large amount of loose talk about the partiality of the committee and its inability to take a calm and judicial view of the matter it was commissioned to investigate. Letters in the local Press demand the appointment of a new committee and a searching inquiry. We have not seen the full report of the Sanitary Committee and therefore do not wish to say anything in regard to the points raised by correspondents in the local papers. The fact, however, that such active interest is taken in the matter speaks well for public interest in hospital management at Hull.

### THE BOLTON INFIRMARY.

It is gratifying to record that during the past week the Bolton Infirmary Committee has received a handsome donation towards the funds of the Institution from Alderman W. Nicholson, J.P., who is the chairman of the Committee and who has always taken a keen practical interest in the work of the hospital with which he has been intimately connected for over forty years. In his letter to the Committee Mr. Nicholson stated that the amount (£1,000) should be placed to the credit of the Endowment Fund, but if Bolton decides that its memorial to the late King should take the form of an extension of the infirmary, the sum should go towards augmenting the Memorial Fund. We are informed that it is not unlikely that such a scheme will be considered by the Mayor, and that the extension will be in the shape of a nurses' home in connection with the infirmary. The Bolton Infirmary has been growing in importance during the last few years and is receiving good support in the neighbourhood, while the local Corporation has intimated its appreciation of the work done by the institution by increasing its annual grant in aid of the funds. Should Alderman Nicholson's suggestion of a memorial home meet with general approval we have no doubt that Bolton will respond to the appeal as generously as subscribers of the North Staffordshire Infirmary have responded to theirs. It is of the utmost importance that all hospital memorials associated with the late King's name should be established clear of debt and endowed in an adequate manner.

### HOSPITAL UNIONISM IN AUSTRALIA.

The Trade Union is rapidly gathering all unto itself in Australia (writes a Melbourne correspondent), the latest innovation being an attempt to form a union of the hospital attendants (men). A meeting in Melbourne of about seventy attendants from the Melbourne, Alfred, Women's, St. Vincent's, and Police Hospitals decided that a union was necessary in view of the "sweating" now perpetrated at all hospitals. It was alleged that seventy hours a week for £4 a month were sometimes worked, and ten hours a day, with only alternate Sundays off, were usual, while those on night duty worked twelve hours with no night off. Provisional officers were appointed to frame rules. The nurses have not yet struck for an eight-hour day, but this will probably follow. The attitude of the men attendants is significant in view of the frequent assertions made by some authorities that Australian hospitals are managed in an exemplary manner. Such hours as are alleged by the strikers to be the average are certainly far in excess of what can be conscientiously demanded of hospital workers, even when we take into consideration the fact that much of the work of hospital attendants is light and scarcely to be ranked as equal to that performed by labourers, for instance.

### MISTAKES IN HOSPITALS.

A good deal of fuss has been made in the Press about the recent unfortunate mistake of a St. Thomas's nurse in administering half an ounce instead of half a drachm of medicine which contained a high percentage of morphia. Those who are acquainted with ward administration know that such mistakes, though they are liable to occur at any time through individual carelessness or oversight, very rarely do take place, and that when they happen it is certainly not the institution or the system that is at fault. Patients in hospital are far safer from overdoses than private patients attended by practitioners who prescribe their own medicines. It is by no means necessary for a nurse to understand a prescription or to be qualified to judge of its toxicity; that is a matter admittedly for the supervision of the hospital pharmacist, just as it is for the average chemist who has a certain responsibility in making up a private recipe, and acquits himself of it by drawing the doctor's attention to any excessive quantity or dosage he may note. The nurse's duty consists merely in administering the medicine in accordance with the written directions on the bed-letter, and we fail to see how any alteration in the present system of sign-writing will enable her to do this better, more accurately, or more safely. However slovenly house physicians may write, there is little likelihood of mistaking even the worst-scribbled ʒj or ʒss for a ℥j or ℥ss. To write these quantities in English would hardly be an advantage, and the English symbols would inevitably suffer mutilation owing to the inherent tendency of the average resident to abbreviate his instructions, so that T.F. may come to mean either tea or tablespoonful. In the case attracting attention at present the mistake was obviously an individual one, discovered the moment after it had been made, although unfortunately it was impossible to obviate its fatal consequences. We know of no similar instance in any of the four great general hospitals of London during the past ten years, and that fact at least speaks volumes for the safety of the present system, when it is borne in mind that during that period nearly half a million in-patient prescriptions, at a moderate estimate, must have been made up and the medicines entrusted to the ward nurses. There is an adequate check upon the nurses, and in some hospitals a medicine such as a tinctus or sleeping mixture is given only in the presence of the night sister. The recommendation added as a rider by the coroner's jury in the St. Thomas's case will not, if carried out, make regrettable oversights less rare than they are at present.

### THIS WEEK'S DRUG MARKET.

There is a distinct improvement in the general tone of business in the drug market, and several articles are in an interesting position. The most noticeable feature is an advance in the price of opium of at least 2s. per lb., following on large sales in the producing district. Morphine is also dearer, and makers of codeine, after making successive reductions in the price, now announce an advance of 10d. per lb. The future of the market in opium and its alkaloids is very uncertain, but the statistical position of opium is such as to lead to the belief that under normal conditions prices should not further advance to any noteworthy extent. Both cream of tartar and tartaric acid still tend dearer. Cod-liver oil is also rather dearer, but the demand at the moment is very small. Buchu leaves seem to have reached their highest value, and sales have been made at prices rather below those recently asked. Ipecacuanha is also tending lower in price. Camomiles are decidedly dearer. It is not unlikely that refiners of glycerin will advance their prices owing to the high value of the crude product. Quicksilver is unchanged in price, but a reduction is not altogether unexpected. Quinine is an extremely dull market, and in consequence of the over-supply of cinchona bark a reduction in price is more probable than an advance, but the price is already so low that a further slight reduction would hardly be noticed by any but large purchasers. Cascara is attracting less attention, but any noticeable demand for the article would in all probability lead to higher prices.

